# Cooperative extension and academic departments partnership: Translating nutrition science messages to diverse audiences

**DOI:** 10.1017/cts.2026.10710

**Published:** 2026-02-18

**Authors:** Olivia Lawler, Gail D’Souza, Travis Masterson, Mary Alice Gettings, Kristina Petersen, Amy Moore, Penny Kris-Etherton

**Affiliations:** 1 Nutritional Sciences, The Pennsylvania State University, USA; 2 Cooperative Extension, The Pennsylvania State University, USA

**Keywords:** Cooperative extension, clinical and Translational Science Institute, educational videos, Dietary guidelines for Americans, spices and Herbs

## Abstract

Cooperative Extension provides research-based outreach to the public, making it a valuable resource for translation scientists. Herein, we describe a collaboration between Cooperative Extension and the Department of Nutritional Sciences at Pennsylvania State University that resulted in widespread dissemination of novel nutrition education materials. Short videos (3 to 5 minutes each) for the public, healthcare professionals and patients focused on using spices and herbs (S&H) to decrease sodium, saturated fat (SFA), and added sugars, and improve diet quality were developed and evaluated. The videos were effective in improving knowledge about S&H, intent to use them, and making healthy diet changes.

## Introduction

The Smith-Lever Act of 1914 established the Cooperative Extension Service, a partnership between the U.S. Department of Agriculture (USDA) and land-grant universities to translate research-based education to the public [1]. With an office in or near most of the 3,000 counties in the U.S., Cooperative Extension is the largest education system of its kind and reaches rural, suburban and urban settings across the socio-economic spectrum. Therefore, Cooperative Extension is well positioned to translate science-based advances across many disciplines to improve public health. Cooperative Extension has been an underutilized partner in public health efforts, though more recently, strategies to improve public health have been outlined through new partnerships and coalitions [2]. A goal of Cooperative Extension’s *National Framework for Health and Wellness* is to increase the number of Americans who are healthy at every life stage [3]. A core mission of this Framework is to serve diverse audiences, including underserved communities with a high prevalence of health disparities.

Translational research is foundational for identifying effective strategies for informing individuals, communities, and populations about new scientific discoveries to improve health.] In 2023, approximately 76.4% of U.S. adults were reported to have one or more chronic conditions/diseases, and 51.4% had multiple chronic conditions [4]. Suboptimal lifestyle behaviors are leading risk factors for chronic diseases and include physical inactivity, tobacco use, excessive alcohol consumption, and poor diet quality [5]. In the U.S., diet quality is suboptimal, with an average population diet quality score of 59 (out of 100), a score that reflects overconsumption of added sugars, SFAs, and sodium, and underconsumption of fruits, vegetables, whole grains, beans, legumes, and seafood [6,7]. Thus, there is a pressing need to identify effective strategies to improve diet quality and other lifestyle behaviors, and reduce chronic disease risk. Clinical and Translational Science Institutes (CTSIs) have been established nationwide to augment the translation of research discoveries to improve population health [8]. A central goal of CTSIs is to form collaborative networks and support coalitions. CTSIs working with Cooperative Extension, offer great synergistic potential for reaching many audiences to improve public health. A notable example of the synergy achieved through a CTSI and Extension partnership is illustrated by the one at Indiana University and Purdue University, which implemented statewide health initiatives and reported improvements in certain health indicators [9]. Other collaborations between CTSIs and Extension are ongoing at the University of Florida, Kansas, Kentucky and New Mexico. Expanding these collaborations to a greater number with additional robust programs offers great potential to impact public health.

The U.S. Dietary Guidelines are the foundation for nutrition guidance to improve health and decrease chronic disease risk [10]. The guidelines have evolved from being nutrient-based to a food-based focus with an emphasis on limiting nutrients of concern, including sodium, SFAs, and added sugars. The 2020-2025 Dietary Guidelines recommend using spices and herbs to flavor foods in place of sodium, SFAs, and added sugars. In response to our previous research demonstrating health benefits of spices and herbs [11], we established a partnership with Penn State Extension and the Department of Nutritional Sciences, and subsequently the College of Medicine, to develop innovative web-based education programs to teach attendees how to improve their diets using S&H. Since Extension typically works with stakeholders and Colleges of Agriculture, our collaboration uniquely built a partnership with new Colleges and Departments. Our outreach strategies were effective for teaching both consumers and healthcare providers how to incorporate S&H into their diets to improve diet quality. This brief report describes the educational programs we implemented and the evaluation results.

## Methods and results

### Developing and evaluating a webinar to teach how to incorporate S&H in recipes to improve diet quality

Faculty and graduate students in the Department of Nutritional Sciences and four Penn State Extension Educators developed a website for the purpose of teaching about using S&H in everyday cooking to reduce sodium, SFAs, and added sugars. The website was used to deliver a 1-hour webinar by Zoom webinar, “Let’s Cook at Home: Herbs and Spices,” which included live cooking demonstrations of five recipes to showcase simple healthy cooking techniques.

Before and after the live webinar, participants were asked to complete a short questionnaire to evaluate their knowledge, confidence, and intention to use S&H. In addition, they were asked if they would be interested in receiving more information about using S&H in their cooking and if short educational videos featuring the recipes would be useful. The change in response (pre- to post-webinar) was evaluated using a Wilcoxon Signed Rank Test to assess median changes. Of the 425 participants who viewed either the live or recorded webinar, 254 individuals watched it live, 171 watched the recording, and 163 participants responded to the survey. The majority of individuals who responded to the survey were female (93%), white (80%) and greater than 55 years old (70%). The demographics of the survey respondents were similar to those who watched the webinar live. Our findings are summarized below:

- 88% reported gaining knowledge in using, pairing, and substituting S&H when preparing meals.
- 88% reported increased confidence in using S&H in their cooking to decrease the amount of sodium, SFAs, and added sugars and increase healthy foods in their diet.
- 88–98% of all participants indicated they would be interested in using S&H to reduce sodium, SFAs, and added sugar and to promote healthier eating.
- 96% reported that three to five-minute videos detailing webinar topics would be useful.

The Penn State Extension website for this program includes educational materials and recipes we developed to support using S&H in cooking and a variety of fact sheets.

Subsequently, five short (3–5 minutes each) videos were developed. Each video featured a recipe using S&H while sharing tips to make cooking easier. The goal of each video was to inspire the participants to use S&H to flavor their food rather than sodium, SFAs, and added sugars.

These five short videos were evaluated by us (described below) and posted on the Center for Nutrition Policy and Promotion (CNPP) USDA Partner Resources website [12]:

- Everyday Salt-Free Seasoning Blend
- Herb Sauced Chicken
- Brown Rice with Herbs and Spices
- Seasoned Roasted Vegetables
- Apple Blueberry Crumble

Three additional webinars and six in-person Let’s Cook with Herbs and Spices Extension workshops were conducted (from July 2023 and August 2024). These workshops were held in-person and online. Because of the success of this program, we conducted further research to evaluate the effectiveness of the nutrition education program in different population groups.

### Studies conducted to evaluate the effects of the videos on knowledge, confidence, and intent to use spices and herbs to improve diet quality

We evaluated the effectiveness of the videos in a cross-section of the U.S. adult population [13]. We utilized a crowd sourcing platform (MTurk)* to assess whether online nutrition education material would increase knowledge about how to use H&S in cooking, confidence in using and intent to use H&S in cooking in a cohort of males and females (ages 18 to 75 years) across the US [14,15]. We evaluated 103 individuals (54.3% males; 45.6% females) who completed all surveys, and diet recalls; the majority of participants were white (84.4%) and not Hispanic or Latino (98%). Participants were given pre- and post-intervention surveys before and after watching the five short videos over the course of approximately one week. In addition, they completed three automated self-administered 24-hour (ASA-24) dietary recalls on two weekdays and one weekend day before and after the intervention to assess changes in diet quality in response to the videos [16]. Each question was based on a 4- or 5-point scale. Survey results were analyzed using Wilcoxon Signed Ranked tests with a priori alpha level set at *P* < 0.05. The Healthy Eating Index-2015 (HEI-2015) was used to assess diet quality since it is the gold standard for comprehensively evaluating diet quality [17]. (HEI-2020 is the same as HEI-2015 for adults.) A description of the methods and results have been reported in detail [13]. We found that short online videos were effective in disseminating nutrition education information (Table 1). Participants reported significant increases in: knowledge gained about using S&H, confidence in using S&H to decrease sodium, SFAs, and added sugars, and intention to use S&H to reduce SFA and sodium. While there was no change in HEI-2015 scores from pre- to post-intervention (52.6 vs 51.9, respectively with no differences in the component scores between groups), there was a significant positive interaction between the change in total HEI-2015 score and participants who had an interest in using S&H to increase healthy foods in their diet. This result indicates that individuals who had a higher interest in using S&H to increase healthy foods were more likely to increase their HEI-2015 score.

**Table 1.** Post-webinar survey results (n = 103)

	Mean change	*P* value
Knowledge of using herbs and spices*	0.1	0.03*
Confidence of using herbs and spices to reduce added sugars**	0.8	<0.001*
Confidence of using herbs and spices to reduce saturated fat**	0.7	<0.001*
Confidence of using herbs and spices to reduce sodium**	0.6	<0.001*
Confidence of using herbs and spices to increase the amount of healthful foods in the diet**	0.6	<0.001*
Intention to use herbs and spices to reduce saturated fat***	0.5	<0.001*
Intention to use herbs and spices to reduce sodium***	0.3	0.01*

*Response Options: 1 to 4; 1 = none and 4 = high.

**Response Options: 1 to 4; 1 = not at all confident and 4 = very confident.

***Response Options: 1 to 5; 1 = strongly disagree and 5 = strongly agree.

With the creation and validation of online educational materials we partnered with family medicine providers to evaluate these materials in three additional cohorts: medical students, medical providers and their patients [18,19]. This process allowed us to understand how educational materials could be distributed and their potential impact on interest, knowledge, confidence, and intent to use S&H in different populations. Medical students (*n* = 50) recruited from the Penn State College of Medicine were 64% female, 64% White, had a mean age of 25 years, and 60% were in their first year of medical school. Medical providers (*n* = 49) recruited from the Family and Community Medicine Department at Penn State Health were 49% male, 65% White, had a mean age of 37.5 years, and 61% were resident physicians [19]. All participants were asked about their interest, knowledge, confidence, and intent in using S&H while preparing a meal both before and after viewing the educational videos. The medical student results showed that all but one measured variable (interest in using S&H to decrease the use of fats high in SFAs) for interest, knowledge, confidence and intent were higher (all *p*-values < 0.05) after watching the videos (Table 2). Similarly, we observed that all measured variables were higher (all *p*-values < 0.05) after watching the videos for medical providers (Table 2). All participants rated the videos as high quality and indicated they would meet the needs of their patients. In addition, medical providers reported value in using educational videos in their clinics.

**Table 2.** Changes in interest, knowledge, confidence and intention to use herbs and spices of medical students, medical providers and patients

Pre–post change scores on the interest, knowledge, confidence, and intention to use herbs and spices questionnaire after viewing the videos from the medical student (*n* = 50) and medical provider (*n* = 49) sample				
	Medical students		Medical providers	
	Mean change	*P* value	Mean change	*P* value
Knowledge in using herbs and spices when preparing foods or a meal*	19.74	<0.0001	14.45	<0.001
Confidence in using herbs and spices to decrease the amount of added sugar used when preparing a meal or food**	26.76	<0.0001	18.71	<0.001
Confidence in using herbs and spices to decrease the amount of fats high in saturated fat (i.e. butter, lard, cream) used when preparing a meal or food**	24.44	<0.0001	20.37	<0.001
Confidence in using herbs and spices to decrease the amount of salt used when preparing a meal or food**	27.22	<0.0001	17.27	<0.001
Confidence in using herbs and spices to increase the palatability and consumption of healthier foods**	17.58	<0.0001	17.25	<0.001
Intention to use herbs and spices as a substitute for saturated fat (i.e. butter, lard, cream) ***	17.16	<0.0001	12.76	<0.001
Intention to use herbs and spices as a substitute for salt***	14.68	<0.0001	8.31	0.004
*Difference in interest, knowledge, confidence, and intent variables between patients after they viewed nutrition education videos vs handouts (n = 102)*				
	Patients			
	Mean change		*P* value	
Knowledge in using herbs and spices in cooking*	4.2		0.26	
Confidence in using herbs and spices to decrease the use of added sugars**	10		0.01	
Confidence in using herbs and spices to decrease the use of saturated fats**	11.3		0.01	
Confidence in using herbs and spices to decrease the use of salt**	11.0		0.003	
Confidence in using herbs and spices to increase palatability and consumption of healthier foods**	4.7		0.19	
Intention to use herbs and spices as a substitute for saturated fats***	13.6		0.001	
Intention to use herbs and spices as a substitute for salt***	13.3		0.001	

*Response Options: 0 to 100; 0 = Very unknowledgeable to 100 = Very knowledgeable.

**Response Options: 0 to 100; 0 = Not confident to 100 = Very confident.

***Response Options: 0 to 100; 0 = Very unlikely to 100 = Very likely.

A similar study was conducted comparing the effectiveness of the educational videos with traditional handout materials when educating patients about the use of S&H to reduce sodium, SFAs, and added sugars during cooking. Participants (*n* = 102) were recruited from Penn State Health family medicine clinics were 61.8% female, 84.3 % White and randomized to either view 5 short videos (52.9%) or read 3 handouts (47.1%) The presurvey was conducted while patients were in the waiting room for their provider appointment, and a post survey was completed on the patients’ smartphones. Participants from the video group had higher scores for interest, knowledge, confidence and intent to use S&H vs. participants who viewed the handouts (Table 2). Patients perceived the videos as clearer (*p* = 0.001) and more appropriately complex (*p* = 0.02) than the handout materials [18].

## Discussion

We describe the effectiveness of short videos in different target groups (e.g., the public, medical students and medical providers, and patients) for translating messages from the 2020–2025 Dietary Guidelines for Americans about using S&H to decrease sodium, SFAs, and added sugars to increase diet quality. A collaboration with Cooperative Extension was valuable in designing and hosting a webinar, Cooking at Home with Herbs and Spices and producing videos that taught principles of using S&H to meet dietary guidelines recommendations. Importantly, all participants really liked learning science-based information via short videos. Being posted on the CNPP website (https://www.myplate.gov/partner-resources), the videos have broad reach. A partnership between Cooperative Extension and translation scientists offers the opportunity to reach many audiences and effectively communicate health messages that benefit all population groups, especially underserved groups.

A report from the Association of Public and Land-Grant Universities, *Healthy Food Systems, Healthy People* [20], calls for strategic partnerships and integration across existing systems including Cooperative Extension and academic departments that also could include CTSIs. Given their shared mission to improve health outcomes, integrating these existing systems that focus on nutrition, health, environment, and agriculture has the potential to expand the reach of all systems and increase the rate by which people receive evidence-based nutrition and health information to improve health outcomes and reduce health disparities [20]. Cooperative Extension, academic departments and CTSIs are positioned to continue to “build bridges” and form strategic partnerships to improve health outcomes in populations across the US, particularly among rural populations who experience health disparities. Based on its extensive reach, Cooperative Extension is positioned to effectively and efficiently deliver evidence-based health and nutrition information through existing community-based and newer online and social media-based delivery models. Communicating health and nutrition information to people in places where they live, work, and socialize is important for the widespread dissemination of current lifestyle recommendations to improve health. Future research needs to focus on identifying effective delivery strategies (and technologies) for reaching the greatest number of individuals with important health messages that are implemented to improve health.
